# Total and high molecular weight adiponectin and ethnic-specific differences in adiposity and insulin resistance: a cross-sectional study

**DOI:** 10.1186/1475-2840-12-170

**Published:** 2013-11-13

**Authors:** Dian C Sulistyoningrum, Danijela Gasevic, Scott A Lear, Joe Ho, Andrew Mente, Angela M Devlin

**Affiliations:** 1 Department of Pediatrics, University of British Columbia, Child and Family Research Institute, 272-950 West 28th Avenue, Vancouver, V5Z 4H4, Canada; 2 Department of Biomedical Physiology and Kinesiology, Simon Fraser University, 8888 University Dr, Burnaby, BC V5A 1S6, Canada; 3 Division of Cardiology, Providence Health Care, Vancouver, Canada; 4 Population Health Research Institute, Hamilton Health Sciences, McMaster University, Hamilton, Canada

**Keywords:** High molecular weight adiponectin, Ethnicity, Insulin resistance, Visceral adipose tissue

## Abstract

**Background:**

Ethnic-specific differences in insulin resistance (IR) are well described but the underlying mechanisms are unknown. Adiponectin is an insulin sensitizing adipocytokine that circulates as multiple isoforms, with high molecular weight (HMW) adiponectin associated with greatest insulin sensitivity. The objective of this study is to determine if plasma total and HMW adiponectin concentrations underlie ethnic-specific differences in IR.

**Methods:**

Healthy Canadian Aboriginal, Chinese, European, and South Asian adults (N = 634) were assessed for sociodemographics; lifestyle; fasting plasma insulin, glucose, and total and HMW adiponectin; and adiposity measures [BMI, waist circumference, waist-to-hip ratio, percent body fat, and subcutaneous and visceral adipose tissue (quantified by computed tomography)]. The homeostasis model assessment-insulin resistance (HOMA-IR) assessed IR.

**Results:**

South Asians had the greatest HOMA-IR, followed by Aboriginals, Chinese, and Europeans (*P* < 0.001). Plasma total and HMW adiponectin concentrations were lower in Chinese and South Asians than Aboriginal and Europeans (*P* < 0.05). Total and HMW adiponectin were inversely associated with HOMA-IR (*P* < 0.001). Ethnicity modified the relationship between HMW adiponectin and HOMA-IR with stronger effects observed in Aboriginals (*P* = 0.001), Chinese (*P* = 0.002), and South Asians (*P* = 0.040) compared to Europeans. This was not observed for total adiponectin (*P* = 0.431). At mean total adiponectin concentrations South Asians had higher HOMA-IR than Europeans (*P* < 0.001).

**Conclusions:**

For each given decrease in HMW adiponectin concentrations a greater increase in HOMA-IR is observed in Aboriginals, Chinese, and South Asians than Europeans. Ethnic-specific differences in HMW adiponectin may account for differences in IR.

## Introduction

Insulin resistance (IR) plays an important role in the etiology of type 2 diabetes [1]. Rates of type 2 diabetes and IR are increasing in certain ethnic populations, such as Aboriginal, Chinese, and South Asian compared to European populations [2-4]. However the underlying mechanisms accounting for these increased rates are unknown.

Adiponectin is an insulin-sensitizing adipocytokine that may contribute to ethnic-specific differences in IR. Circulating adiponectin concentrations are inversely proportional to adiposity [5], and adiponectin circulates as multiple isoforms characterized as low, medium, and high molecular weight (HMW), with the HMW isoform being the most abundant and demonstrating the greatest insulin sensitizing properties [6,7].

Few studies have explored ethnic-specific differences in the relationship between adiponectin and IR and the findings have been inconsistent. One investigation reported no relationship in South Asian and European South Africans [8]. Whereas others observed a stronger relationship between total and HMW adiponectin concentrations and IR in Europeans than in South Asians [9] and a stronger relationship in South Asian and Aboriginals compared to Chinese and Europeans [10]. However, these studies only considered surrogate measures of adiposity, such as BMI or waist circumference, when assessing the relationship between adiponectin and IR, which may account for the discrepant findings. Further studies are required that consider body fat distribution, especially visceral adipose tissue (VAT), given that VAT is strongly associated with IR [11] and VAT deposition differs according to ethnicity [12].

Furthermore, despite the evidence of ethnic-specific differences in body fat distribution [12] little is known regarding ethnic-specific differences in the relationship between adiposity and adiponectin. Most prior studies have only considered BMI and waist circumference [10,13,14]. Direct measures of adiposity have rarely been utilized and were predominantly explored in African, Caucasian, and Hispanic American populations [15,16]. Less is known about the relationship between adiposity and adiponectin in other ethnic groups like Aboriginals, Chinese, and South Asians and whether it differs from that of Europeans.

The objectives of this study are: i) to explore ethnic-specific differences in the relationship between adiposity (body composition and body fat distribution) and plasma total and HMW adiponectin concentrations; and ii) to investigate whether ethnicity modifies the relationship of plasma total and HMW adiponectin concentrations with IR independently of visceral adiposity in a multi-ethnic population of Aboriginals, Chinese, Europeans, and South Asians.

## Methods

### Study participants and assessments

Study participants were recruited as part of the Multicultural-Community Health Assessment Trial (M-CHAT) [17]. As previously described [12], the cohort includes apparently healthy men and women (aged 30 to 65 years old) who were of exclusive European (continental Europe, Ireland, and the United Kingdom), Chinese (China, Hong Kong, and Taiwan), Aboriginal (reserve and non-reserve residents), or South Asian (Bangladesh, India, Nepal, Pakistan, and Sri Lanka) descent living in and around Vancouver, Canada. Only those study participants of Aboriginal descent with at least 3 grandparents of exclusive Aboriginal origin were included in the study due to high prevalence of mixed ethnic origins in Aboriginal populations. At the time of recruitment and enrolment in the study, participants had no known cardiovascular disease or type 1 or 2 diabetes diagnoses and must have had a stable body weight in the past 3 months (no more than 2 kg of body weight lost or gained). For non-Aboriginals, immigrants (who have lived in Canada for more than 3 years to allow for acculturation) or first or second generation Canadians were included. The recruitment for study participants was done in such a way that there is an equal representation of participants with healthy, overweight, and obese BMI in each ethnic group. The study was approved by the University of British Columbia, Children and Women’s Health Centre of British Columbia, Providence Health Care, and Simon Fraser University Research Ethics Boards. All study participants provided informed consent.

Participants were assessed for age, sex (self-report), sociodemographics (education and income levels), family history of type 2 diabetes, and lifestyle factors (smoking status). Total daily energy intakes were quantified by collecting a 3-d food record and analyzed by a registered dietitian using commercially available software (Food Processor SQL software, ESHA, Salem, OR). Leisure-time physical activity was assessed as the average minutes per week of activity over the previous year (self-report) as previously described [12].

### Assessment of insulin resistance

Insulin resistance was calculated using the previously validated homeostasis model assessment of insulin resistance (HOMA-IR) [18]. Fasting plasma glucose concentrations were determined by a glucose hexokinase II method using an ADVIA 1650 analyzer (Bayer Health Care, Morristown, NJ). Fasting plasma insulin concentrations were quantified by a commercial ELISA assay using Immulite 2500 analyzer (Diagnostic Products, Los Angeles, CA). Coefficients of variations for intra- and inter-assay precision for were less than 7.4%.

### Adiposity and body fat distribution

Body mass index was calculated by body weight (kg)/height (m)^2^. Waist circumference (WC) was measured by taking the average of two readings at the point of maximal narrowing. Hip circumference was recorded as the mean of two measures taken at the point of maximal gluteal protuberance from the lateral view over undergarments. Waist/hip ratio (WHR) was calculated by dividing WC by hip circumference. Total body fat was measured by dual-energy X-ray absorptiometry with a Norland XR-36 scanner (Norland Medical Systems, White Plains, NY). The percentage of total body fat was calculated by dividing total body fat by total body mass. A computed tomography scan was used to take a single 10 mm-thick cross-sectional slice at the lumbar 4/5 (L4/L5) of the inter-vertebral disc to quantify total abdominal adipose tissue and VAT. Surface area of the scan was read using the CTi Advantage Scanner (GE, Milwaukee, WI) and the computation of surface areas were performed using SliceOmatic v.4.2. (Tomovision, Montreal). Total abdominal adipose tissue is defined as the total area in cm^2^ within an attenuation range of (-190) to (-30) Hounsfield units for adipose tissue in the L4/L5 cross-sectional slice image. VAT is the total area within the aforementioned attenuation range that falls within the abdominal wall. Subcutaneous adipose tissue (SAT) was calculated as the difference between total abdominal adipose tissue and VAT.

### Quantification of plasma adiponectin concentrations

Plasma total and HMW adiponectin concentrations were quantified using a commercially available ELISA (ALPCO Diagnostics, Salem, NH). HMW adiponectin concentrations were quantified by pretreating plasma with proteinase K, which selectively digests low and medium molecular weight adiponectin. The inter-assay (between plates) coefficient of variability (CV) was 6.15% and the intra-assay CV was 2.4%.

### Statistical analyses

Of the 822 potentially available M-CHAT study participants, we excluded 188 because of lack of information on HOMA-IR, total- or HMW adiponectin, which left 634 individuals for our analyses. Distribution of continuous variables was explored for normality, and variables that were not normally distributed (HOMA-IR, total- and HMW-adiponectin, VAT, SAT, physical activity) were natural logarithm (ln) transformed. Continuous variables are presented as means (95% confidence interval [CI]) or geometric means (95% CI) if not normally distributed. Ethnic differences in categorical variables were explored using Chi-square test. The difference in continuous variables was investigated by analysis of variance (ANOVA) or general linear modeling where the analyses were controlled for age and sex. Post hoc pairwise comparisons were performed using Bonferroni tests to adjust for multiple comparisons.

Linear regression models were used to determine the relationship between adiposity measures (BMI, WC, waist/hip ratio, percent total body fat, SAT and VAT; independent variables) and total- and HMW- adiponectin (dependent variables). Separate models were used for each adiposity variable and each of the adiponectin variables, while adjusting for covariates (age, sex, ethnicity, education, smoking status, physical activity, and total dietary energy intake). To explore whether ethnicity modifies the relationship between adiposity and adiponectin, we investigated the interactions of ethnicity with each of the adiposity measures. We found that ethnicity did not modify the relationship of adiposity with either total adiponectin or HMW adiponectin (p > 0.05 for all ethnicity by adiposity interactions). Furthermore, given the sex differences in body fat accumulation, we additionally explored whether sex modifies the relationship of adiposity with total and HMW adiponectin. Given that all interactions were not significant (p > 0.05 for all), the relationship between adiposity and adiponectin was explored on a total sample.

The relationship between total and HMW adiponectin (independent variables) and HOMA-IR (dependent variable) was examined using linear regression. We explored interactions of ethnicity with both total and HMW adiponectin in order to explore whether ethnicity modifies the relationship between adiponectin and IR. While ethnicity was found not to modify the association between total adiponectin and IR (ethnicity by total adiponectin interaction, p = 0.431), the ethnicity by HMW adiponectin interaction was found significant (p = 0.010). Therefore, the relationship between total adiponectin and IR was explored on a total sample after adjusting for ethnicity and other covariates of interest (age, sex, family history of diabetes mellitus, education, smoking status, physical activity, total dietary energy intake, BMI and WHR). Ethnic specific differences in the relationship between HMW adiponectin and IR were also explored using linear regression including the interaction term and adjusting for the above-mentioned covariates. To evaluate whether the association of total- and HMW- adiponectin concentrations with HOMA-IR was independent of VAT, models were additionally adjusted for VAT.

All regression models were checked for multicolinearity; variance inflation indices and tolerance statistics were well below 10 and above 0.2, respectively, indicating that there was no collinearity in our data. Statistical analyses were performed using Statistical Package for Social Sciences (IBM SPSS version 19). Results are considered significant if *p* < 0.05 (two-sided).

## Results

### Study participant characteristics

Significant ethnic-specific differences in age, education, current smoking, physical activity, dietary energy intake, measures of adiposity, plasma glucose concentrations, and HOMA-IR were observed (Table 1). Specifically, Chinese and Europeans were significantly older, had a higher level of formal education, and higher dietary total energy intake compared to Aboriginals and South Asians. Furthermore, Aboriginals and Europeans were more likely to be current smokers, but engage in greater physical activity than Chinese and South Asians. Aboriginals had significantly higher BMI, WC, and WHR compared to the other ethnic groups, whereas Chinese participants had the lowest BMI and WC. Percent body fat, VAT, SAT, plasma insulin concentrations, and HOMA-IR were significantly hig-her among Aboriginals and South Asians compared to Chinese and Europeans (*P* < 0.05 for all).

**Table 1:** Characteristics of study participants by ethnic group

	**Aboriginal (n = 142)**	**Chinese (n = 163)**	**European (n = 164)**	**South Asian (n = 165)**	**Overall *p *** **value**
Age (years)	45.7 (44.4-47.1)	48.1 (46.7-49.4)	51.1 (49.8-52.4)	45.1 (43.8-46.4)	< 0.001 ^b, d, e, f^
Females	75 (52.8%)	90 (55.2%)	78 (47.6%)	83 (50.3%)	0.550
Education					< 0.001
High school or less	55 (38.7%)	53 (32.5%)	33 (20.1%)	68 (41.2%)	
More than high school	87 (61.3%)	110 (67.5%)	131 (79.9%)	97 (58.8%)	
Current smoker	41 (28.9%)	6 (3.7%)	13 (7.9%)	6 (3.6%)	< 0.001
Physical activity (min/week) †*	288.3 (240.6-345.5)	162.1 (136.6-192.5)	247.6 (208.9-293.5)	145.5 (122.6-172.4)	< 0.001 ^a, c, d, f^
Diet (total energy, kcals) †	1821 (1725–1918)	2010 (1923–2097)	2100 (2012–2188)	1755 (1665–1845)	< 0.001 ^a, b, e, f^
BMI (kg/m^2^)†	29.2 (28.4-29.9)	25.7 (25.0-26.4)	27.7 (27.0-28.4)	27.7 (27.1-28.4)	< 0.001 ^a-e^
WC (cm) †	95.7 (94.0-97.4)	83.5 (81.9-90.8)	89.2 (87.6-90.8)	88.7 (87.1-90.3)	< 0.001 ^a-e^
WHR†	0.94 (0.93-0.95)	0.88 (0.87-0.88)	0.87 (0.86-0.88)	0.89 (0.88-0.90)	< 0.001 ^a-c^
Percent body fat (%)†	35.0 (33.9-36.0)	30.8 (29.8-31.7)	31.9 (31.0-32.9)	36.4 (35.4-37.4)	< 0.001 ^a, b, e, f^
VAT (cm^2^)†*	117.6 (109.1-126.8)	92.4 (86.2-99.1)	95.8 (89.2-102.8)	118.5 (110.5-127.1)	< 0.001 ^a, b, e, f^
SAT (cm^2^)†*	308.3 (287.4-330.6)	212.3 (199.1-226.6)	260.1 (243.5-277.8)	299.8 (280.9-319.9)	< 0.001 ^a, b, d-f^
Glucose (mmol/L) †*	5.25 (5.15-5.35)	5.27 (5.18-5.37)	5.10 (5.01-5.20)	5.35 (5.25-5.45)	< 0.001 ^f^
Insulin (μU/mL) †*	72.60 (66.35-79.51)	60.70 (55.76-66.02)	57.69 (52.93-62.87)	77.71 (71.38-84.61)	< 0.001 ^a, b, e, f^
HOMA-IR†*	2.44 (2.21-2.70)	2.05 (1.86-2.25)	1.88 (1.71-2.07)	2.66 (2.42-2.92)	< 0.001 ^b, e, f^
Total adiponectin (μg/mL) †*	5.17 (4.80-5.56)	4.15 (3.88-4.45)	5.18 (4.83-5.56)	4.41 (4.12-4.73)	< 0.001 ^a, c, d, f^
HMW adiponectin (μg/mL) †*	2.45 (2.18-2.75)	1.59 (1.43-1.77)	2.35 (2.11-2.62)	1.84 (1.65-2.05)	< 0.001 ^a, c, d, f^

Continuous data presented as mean (95% CI). Categorical data presented as n (%).

†Means are adjusted for age and sex. *Geometric means (95% CI).

The overall p-value: ethnic differences in continuous and categorical variables were explored by ANOVA and Chi-square tests, respectively. Post hoc pairwise comparisons: ^a^p < 0.05, Aboriginal vs. Chinese, ^b^p < 0.05, Aboriginal vs. European, ^c^p < 0.05, Aboriginal vs. South Asian, ^d^p < 0.05, Chinese vs. European, ^e^p < 0.05, Chinese vs. South Asian, ^f^p < 0.05, European vs. South Asian.

### Adiponectin, ethnicity, and insulin resistance

Total and HMW adiponectin concentrations were similar in Aboriginals and Europeans and significantly higher compared to Chinese and South Asians (Table 1). Total and HMW adiponectin concentrations were significantly inversely associated with all measures of adiposity (BMI, WC, WHR, percent body fat, VAT, and SAT) after adjustments for age, sex, ethnicity, education, smoking status, physical activity and total dietary energy intakes (Table 2). The strongest relationships were observed between WHR and ln total adiponectin [B (95% CI): -2.118 (-2.714, -1.523), *P* < 0.001], and between WHR and ln HMW adiponectin concentrations [B (95% CI): -2.663 (-3.609, -1.717), *P* < 0.001].

**Table 2:** Association between adiposity and adiponectin

	**ln total adiponectin**			**ln HMW adiponectin**		
	**B (95% CI)**	**Standardized B**	**p value**	**B (95% CI)**	**Standardized B**	***p *** **value**
BMI	-0.021 (-0.030, -0.013)	-0.191	< 0.001	-0.035 (-0.048, -0.022)	-0.199	< 0.001
WC	-0.011 (-0.014, -0.007)	-0.259	< 0.001	-0.017 (-0.023, -0.011)	-0.255	< 0.001
WHR	-2.118 (-2.714, -1.523)	-0.385	< 0.001	-2.663 (-3.609, -1.717)	-0.305	< 0.001
% BF	-0.007 (-0.013, -0.001)	-0.136	0.019	-0.011 (-0.021, -0.002)	-0.134	0.019
ln VAT	-0.267 (-0.350, -0.184)	-0.264	< 0.001	-0.339 (-0.471, -0.208)	-0.210	< 0.001
ln SAT	-0.120 (-0.209, -0.031)	-0.111	0.008	-0.225 (-0.363, -0.086)	-0.131	0.002

All linear regression models were adjusted for age, sex, ethnicity, formal level of education, smoking status, physical activity, and energy intake.

B, beta-value; BMI, body mass index; % BF, percent body fat; SAT, subcutaneous adipose tissue; VAT, visceral adipose tissue; WC, waist circumference; WHR, waist-to-hip ratio.

**Figure 1:**
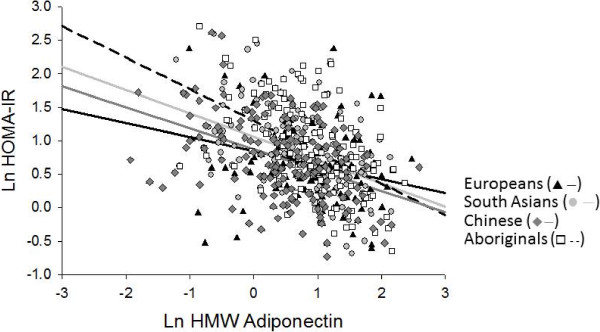
**Interaction between plasma HMW adiponectin concentrations and ethnicity on HOMA-IR.** The relationship between HMW-adiponectin on HOMA-IR in Aboriginals (dashed line), Chinese (solid dark grey line) and South Asians (solid light grey line) is different from that of Europeans (solid black line).

A significant inverse association between plasma total adiponectin concentration and HOMA-IR was found after adjustment for age, sex, ethnicity, family history of diabetes mellitus, education, smoking status, physical activity, dietary total energy intake, BMI, and WHR (Model 1) (Table 3). In this model, ethnicity had a significant effect on HOMA-IR. Specifically, HOMA-IR was significantly higher in South Asians compared to Europeans [B (95% CI): 0.203 (0.087, 0.318), *P* = 0.001]. When the model was further adjusted for VAT (Model 2, Table 3), the relationship between plasma total adiponectin concentration and HOMA-IR was slightly attenuated but remained significant [B (95% CI): -0.300 (-0.385, -0.214), *P* < 0.001]. The effect of ethnicity on HOMA-IR was also attenuated with inclusion of VAT in the model; however, HOMA-IR remained significantly higher in South Asians compared to Europeans [B (95% CI): 0.155 (0.044, 0.266), *P* = 0.006] (Model 2, Table 3). While the association between total adiponectin and HOMA-IR was independent of ethnicity; ethnicity was found to modify the relationship between plasma HMW adiponectin concentration and HOMA-IR (Figure 1). Compared to Europeans, the effect of HMW-adiponectin on HOMA-IR was significantly different in Aboriginals (B (95% CI) = -0.238 (-0.391, -0.086), *P* = 0.002), Chinese (B (95% CI) = -0.199 (-0.328, -0.069), *P* = 0.003, and South Asians (B (95% CI) = -0.146 (-0.285, -0.008), *P* = 0.038). The addition of VAT did not substantially change the modifying effect of ethnicity on the relationship between HMW adiponectin and HOMA-IR. Namely, after additional adjustment for VAT, the effect of HMW-adiponectin on HOMA-IR in Aboriginals (B (95% CI) = -0.236 (-0.381, -0.092), *P* = 0.001), Chinese (B (95% CI) = -0.195 (-0.318, -0.072), *P* = 0.002), and South Asians (B (95% CI) = -0.137 (-0.268, -0.006), *P* = 0.040) was still significantly different from that of Europeans.

**Table 3:** Association between total adiponectin and HOMA-IR

**Outcome: in HOMA-IR**	**Total adiponectin**	
	**B (95%CI)**	***p *** **value**
Model 1		
ln Total adiponectin	-0.331 (-0.421, -0.242)	< 0.001
Ethnicity		
Aboriginal vs. European	0.001 (-0.126, 0.125)	0.995
Chinese vs. European	0.061 (-0.052, 0.174)	0.287
South Asian vs. European	0.203 (0.087, 0.318)	0.001
BMI	0.054 (0.043, 0.064)	< 0.001
WHR	2.280 (1.530, 3.031)	< 0.001
Model 2		
ln Total adiponectin	-0.300 (-0.385, -0.214)	< 0.001
Ethnicity		
Aboriginal vs. European	0.022 (-0.098, 0.141)	0.722
Chinese vs. European	0.061 (-0.046, 0.169)	0.264
South Asian vs. European	0.155 (0.044, 0.266)	0.006
BMI	0.030 (0.018, 0.041)	< 0.001
WHR	1.251 (0.488, 2.013)	0.001
VAT	0.473 (0.351, 0.594)	< 0.001

Outcome variable is ln HOMA-IR. Model 1 is adjusted for ethnicity, age, sex, family history of diabetes, formal level of education, BMI, WHR, smoking status, physical activity and energy intake. Model 2 = Model 1 + VAT.

BMI, body mass index; VAT, visceral adipose tissue; WHR, waist-to-hip ratio.

## Discussion

The current study was designed to explore ethnic-specific differences in the relationship between direct measures of adiposity and plasma total and HMW adiponectin concentrations in a multi-ethnic population of Aboriginals, Chinese, Europeans, and South Asians. We further determined whether ethnicity modifies the relationship of plasma total and HMW adiponectin concentrations with IR, independent of visceral adiposity. We report that all measures of adiposity, surrogate (BMI, WC, WHR) and direct (percent body fat, SAT, and VAT), are inversely associated with plasma total and HMW adiponectin concentrations but that ethnicity has no effect on these relationships. We also found that plasma total and HMW adiponectin concentrations are inversely associated with HOMA-IR and that inclusion of VAT in the model only slightly attenuated the relationship. Most interestingly, we found that ethnicity modified the relationship between HMW adiponectin and IR in that for every decrease in HMW adiponectin concentration a greater increase in HOMA-IR was observed in Aboriginals, Chinese, and South Asians than in Europeans. This ethnic-specific effect was not observed for the relationship between total adiponectin and IR.

Adiponectin is an insulin-sensitizing adipocytokine that may be a mediating factor that underlies ethnic-specific differences in IR. Prior studies by others have been inconclusive [8-10,19,20] and little is known regarding the role of body composition and body fat distribution in this relationship. We have previously reported that South Asians and Aboriginals have significantly higher VAT deposition compared to Chinese and Europeans [12] and this is accompanied by ethnic-differences in IR [21]. Most importantly, in the current study, we found that ethnicity modifies the relationship between plasma HMW adiponectin concentrations and HOMA-IR, in that for each given decrease in plasma HMW adiponectin concentration, a greater increase in HOMA-IR was observed in Aboriginals, Chinese, and South Asians compared to Europeans. The same interaction was not observed for plasma total adiponectin concentrations. Taken together our findings suggest that HMW adiponectin may play a role in the ethnic-specific differences in IR, which was previously attributed, in part, to ethnic-specific differences in VAT deposition. Furthermore, the differences in HMW adiponectin concentrations may also account for differences in diabetes severity and cardiovascular complications observed among different indigenous populations [22].

As has been reported by others, our findings that ethnicity had no effect on the relationship between plasma total and HMW adiponectin concentrations and surrogate measures of adiposity (BMI, WC, WHR), percent body fat, and body fat distribution (VAT and SAT) is in contrast to the findings of another Canadian study that reported an inverse relationship between surrogate measures of adiposity and plasma total adiponectin concentrations in Aboriginals, Chinese, and Europeans but not in South Asians [10]. However, similar to our findings, an inverse relationship between serum total adiponectin concentrations and VAT was reported in a population of South Asians living in California [19].

Adipose tissue is an endocrine organ that releases a number of adipocytokines, such as leptin and adiponectin, which play important roles in appetite regulation and cellular energy metabolism [11]. In addition, adiponectin has insulin-sensitizing functions and may contribute to ethnic-specific differences in IR. Similar to what we report here, another group reported that plasma total and HMW adiponectin concentrations were also lower in South Asians compared to Europeans but an inverse relationship with IR was only observed in Europeans and not in South Asians [9]. Lower total adiponectin concentrations were also reported in a population of South Asians compared to African or Chinese subjects from Trinidad [20]. In our current study we report the opposite effect, with the strongest relationship between plasma HMW adiponectin concentrations and IR found in Aboriginals, Chinese, and South Asians compared to Europeans. Interestingly, we did not find an ethnic-specific effect on the relationship between plasma total adiponectin concentrations and IR. This is in contrast to the findings of another group that did report an effect of ethnicity on the inverse relationship between plasma total adiponectin concentrations and IR with South Asians and Aboriginals showing the strongest relationship compared to Europeans and Chinese [10].

The role of adiponectin in the maintenance of glucose-insulin homeostasis has been investigated in the past two decades. A study in isolated epididymal rat adipocytes incubated with globular recombinant adiponectin reported that adiponectin facilitated insulin-stimulated glucose uptake via the activation of AMP-kinase pathway [23]. A study in male C57BL/6 J, *ob/ob,* and female non-obese diabetic mice showed that an injection with 84 μg/g body weight of recombinant full-length adiponectin reduced serum glucose concentrations and subsequently reduced hyperglycemia in the male *ob/ob* and female NOD mice [24]. In the same study, it was also reported that the presence of 50 μg/mL of full-length adiponectin on isolated primary hepatocytes increased the ability of insulin to suppress glucose production. Furthermore, variants in the gene encoding one of the adiponectin receptors (*ADIPOR2*) were reported to be associated with increased risk of developing type 2 diabetes and greater cardiovascular disease risk factors in individuals with glucose intolerance [25].

In the current study we also considered body fat distribution and specifically we were interested in investigating the interplay between adiponectin and VAT as predictors of HOMA-IR because of the evidence supporting a strong inverse association between adiponectin and VAT [26-28]. A few studies have investigated whether adiponectin synthesis and secretion is influenced by body fat location. A study in adipose tissue explants obtained from subjects undergoing abdominal surgery reported that total adiponectin secretion by adipocytes from SAT explants was lower in obese than in non-obese subjects but this was not observed for adipocytes from VAT explants [29]. These findings suggest that adiponectin secretion by adipose tissue may depend on body fat location. In line with this, we report that VAT has a stronger inverse association with plasma total and HMW adiponectin concentrations than SAT. In agreement with our findings, a study in cultured adipocytes from human omental (VAT) adipose tissue and SAT reported that adiponectin secretion by the omental adipocytes was lower than that by SAT adipocytes [30]. This study also reported that adiponectin secretion by the omental adipocytes was increased in the presence insulin, an effect that was but not observed in adipocytes from SAT, further supporting a role for adiponectin and VAT in IR.

### Study limitations

Our study has some limitations. We used HOMA-IR to assess IR, which is inferior to the euglycemic-hyperinsulinemic clamp method to assess IR. However, the use of HOMA-IR as a surrogate marker of IR is less invasive and more feasible in large population studies [18]. Furthermore, given our study is cross-sectional in design we cannot determine the causal-effect of adiponectin on HOMA-IR. Despite these limitations, our study has strengths, which include our elaborate assessment of adiponectin by quantifying plasma total and HMW adiponectin concentrations. This is important because HMW adiponectin is suggested to be the most abundant and have the greatest insulin-sensitizing properties [6,7]. Furthermore, we included direct measures of body fat distribution (VAT) in our models, which is more strongly associated with IR compared to other measures of adiposity [11]. Other limitations of our study are the differences in age range and the relatively small sample size of the different ethnic groups.

In summary, the relationship between plasma HMW adiponectin and HOMA-IR is influenced by ethnicity with the greater effect observed for Aboriginals, Chinese, and South Asians compared to Europeans. Even after adjustment for VAT, the association between adiponectin and HOMA-IR remained significant, suggesting that adiponectin plays a role in IR across different ethnic groups independent of VAT.

## Abbreviations

ANOVA: Analysis of variance; CI: Confidence interval; HOMA-IR: Homeostasis model assessment of insulin resistance; HMW: High molecular weight; IR: Insulin resistance; M-CHAT: Multicultural-community health assessment Trial; SAT: Subcutaneous adipose tissue; VAT: Visceral adipose tissue; WC: Waist circumference; WHR: Waist-hip ratio.

## Competing interests

The authors declare no conflicts of interest to disclose from any of the authors.

## Authors’ contributions

SAL and AMD conceived and designed the research. DCS, JH, and SAL performed the research. DCS, DG, and AMD analyzed the data. DCS, DG, and AMD wrote the manuscript. SAL and AMD had primary responsibility for final content. All authors read and approved the final manuscript.
